# Betulinic acid chemosensitizes breast cancer by triggering ER stress-mediated apoptosis by directly targeting GRP78

**DOI:** 10.1038/s41419-018-0669-8

**Published:** 2018-05-25

**Authors:** Youli Cai, Yifeng Zheng, Jiangyong Gu, Shengqi Wang, Neng Wang, Bowen Yang, Fengxue Zhang, Dongmei Wang, Wenjun Fu, Zhiyu Wang

**Affiliations:** 1 Integrative Research Laboratory of Breast Cancer, The Research Centre of Integrative Medicine, Discipline of Integrated Chinese and Western Medicine & The second affiliated Hospital of Guangzhou University of Chinese Medicine, Guangzhou, Guangdong China; 2 College of Basic Medicine, Guangzhou University of Chinese Medicine, Guangzhou, Guangdong China; 3 Department of Mammary Disease, Guangdong Provincial Hospital of Chinese Medicine, Guangzhou, Guangdong China; 4 Post-Doctoral Research Center, Guangzhou University of Chinese Medicine, Guangzhou, Guangdong China; 5 School of Pharmaceutical Sciences, Sun Yat-Sen University, Guangzhou, Guangdong China

## Abstract

Stress-induced cellular defense machinery has a critical role in mediating cancer drug resistance, and targeting stress-related signaling has become a novel strategy to improve chemosensitivity. Betulinic acid (BA) is a naturally occurring pentacyclic triterpenoid with potent anticancer bioactivities in multiple malignancies, whereas its underlying mechanisms remain unclear. Here in, we found that BA has synergistic effects with taxol to induce breast cancer cells G2/M checkpoint arrest and apoptosis induction, but had little cytotoxicity effects on normal mammary epithelial cells. Drug affinity responsive target stability (DARTS) strategy further identified glucose-regulated protein 78 (GRP78) as the direct interacting target of BA. BA administration significantly elevated GRP78-mediated endoplasmic reticulum (ER) stress and resulted in the activation of protein kinase R-like ER kinase (PERK)/eukaryotic initiation factor 2a/CCAAT/enhancer-binding protein homologous protein apoptotic pathway. GRP78 silencing or ER stress inhibitor salubrinal administration was revealed to abolish the anticancer effects of BA, indicating the critical role of GRP78 in mediating the bioactivity of BA. Molecular docking and coimmunoprecipitation assay further demonstrated that BA might competitively bind with ATPase domain of GRP78 to interrupt its interaction with ER stress sensor PERK, thereby initiating the downstream apoptosis cascade. In vivo breast cancer xenografts finally validated the chemosensitizing effects of BA and its biofunction in activating GRP78 to trigger ER stress-mediated apoptosis. Taken together, our study not only uncovers GRP78 as a novel target underlying the chemosensitizing effects of BA, but also highlights GRP78-based targeting strategy as a promising approach to improve breast cancer prognosis.

## Introduction

Breast cancer is one of the most common malignancies affecting women worldwide. In 2012, it was estimated that there were 1.67 million new cases and 521,900 breast cancer-related deaths around the world^1^. Although great strides have been made in breast cancer therapy, chemotherapy still remains the cornerstone against both early-stage and advanced breast cancers^2^. Meanwhile, it cannot be ignored that chemoresistance, whether inherent or acquired, has become one of the most common challenges in breast cancer treatment. Approximately 40% of all breast cancer patients confront chemotherapy failure and recurrence, which is closely correlated with poor chemosensitivity^3^. More importantly, development of efficacious drug resistance inhibitors has been slow and disappointing due to increased cytotoxicity effects, off-targets properties, and poor cancer tissue distribution^4^. Therefore, there is an urgent need to develop more effective and safer strategies that increase breast cancer chemosensitivity and improve prognosis.

In the past decade, various factors have been intensively investigated to reveal the underlying mechanisms of cancer drug resistance, such as drug transporters, cancer cell heterogeneity, autophagy induction, oxidative stress, and target mutation^5^. Apart from these mechanisms, a key factor is the cells’ capacity to handle the stress induced by chemotherapy agents. During malignant evolution, cells develop efficient defense machinery that allows them to adapt to harmful stresses including hypoxia, nutrient deprivation, oncogenic signaling, and the toxic events induced by chemotherapy agents^6,7^. Stress is considered a critical filter that promotes cancer evolution and generation of chemoresistant cancer cells^8,9^. For example, administration of chemotherapy agents has been demonstrated to be an efficient strategy to enrich cancer stem cells^10,11^. Meanwhile, autophagy inhibition has been validated to increase cancer chemosensitivity in multiple malignancies^12,13^. Furthermore, a reactive oxygen species (ROS)-scavenging system has become one of the significant mechanisms contributing to chemoresistance development. For example, the antioxidant protein Nrf2 was found to be significantly upregulated in chemoresistant cancer cells, and Nrf2 silencing was validated to promote cancer chemosensitivity to doxorubicin, cisplatin, and etoposide^14,15^. More importantly, endoplasmic reticulum (ER) stress is emerging as a significant mechanism for chemoresistance by degrading damaged proteins through proteasome. Therefore, targeting stress-related modulators has become a promising strategy to improve cancer chemosensitivity^8,9^.

Upon chemotherapy induction, unfolded proteins significantly accumulate in the cytoplasm, resulting in the activation of ER stress, a process known as unfolded protein response (UPR)^16,17^. ER stress is coordinated by three types of sensors including inositol-requiring transmembrane kinase/endoribonuclease 1a, activating transcription factor 6, and protein kinase R-like ER kinase (PERK), which are maintained in an inactive state by binding with the chaperone glucose-regulated protein 78 (GRP78; also known as binding immunoglobulin protein, BiP) under normal conditions^18^. Once UPR is activated, GRP78 is disassociated from the sensors and preferentially binds to the unfolded or misfolded proteins, leading to their proteasomal degradation. Therefore, transient ER stress activation can promote cancer cell escape from damaging events and facilitate drug resistance, and ultimately, metastasis. However, persistent ER stress will activate PERK downstream signaling eukaryotic initiation factor 2a, which subsequently inhibits protein translation and activates the transcription factor CCAAT/enhancer-binding protein homologous protein (CHOP) to induce apoptosis^19,20^. Therefore, ER stress-induced apoptosis might be a potential strategy to enhance chemosensitivity.

Candidate drugs for enhancing chemosensitivity ideally should be selective, potent, and relatively non-toxic. Betulinic acid (BA) is a pentacyclic triterpenoid principally isolated from the bark of the white birch (*Betula pubescens*)^21^. BA exhibits significant antitumor activities on various kinds of cancer cells, including breast cancer^22^. Mechanistic studies have revealed that BA can trigger apoptosis induction in multiple cancer cell lines, and thus inhibit cancer growth and exert synergistic effects with chemotherapy agents. Meanwhile, several reports also found that BA could suppress cancer angiogenesis, invasion, and cancer stem cells^23,24^. A recent study also indicated that the antitumor activities of BA were closely associated with degradation of the transcription factor Sp1^25,26^. However, the direct molecular target of BA and its underlying mechanisms involved in apoptosis induction are still unknown.

In the current study, we found that BA synergistically increases taxol chemosensitivity via apoptosis induction and cell cycle arrest at the G2/M checkpoint. Drug affinity responsive target stability (DARTS) technique further identified the precise target of BA as GRP78. Molecular docking and mechanism studies further validated that BA-induced ER stress-mediated apoptosis by interrupting the interaction between PERK and GRP78. In vivo breast cancer xenografts finally confirmed the GRP78-mediated chemosensitizing effects of BA. Taken together, our study not only provides a promising candidate to enhance chemosensitivity, but also sheds novel light on utilizing GRP78 to design a chemosensitizing strategy in the future.

## Results

### BA represses breast cancer cell proliferation and augments chemosensitivity of taxol

**Fig. 1:**
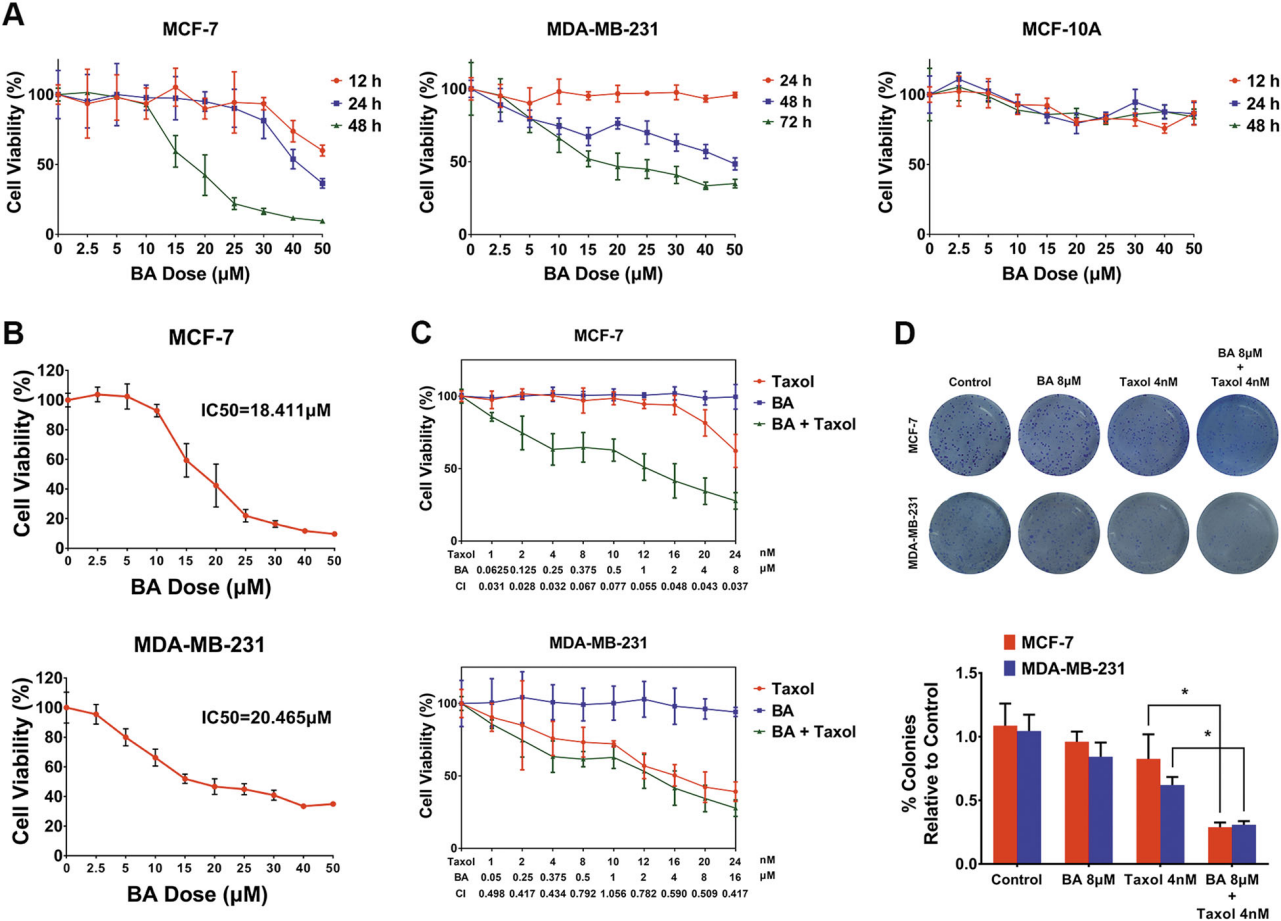
BA inhibits breast cancer cells proliferation and aggravates the inhibitory effects of taxol. **a** MCF-7, MDA-MB-231, and MCF-10A cells were treated with BA at concentrations ranging from 0 to 50 μM time dependently. Cell viability was assessed by CCK-8 assays. BA inhibited growth in breast cancer cells MCF-7 and MDA-MB-231 dose- and time dependently, but had minimal inhibitory effect on normal mammary epithelial cells MCF-10A. **b** The half inhibitory concentration (IC_50_) of BA on MCF-7 at 48 h and MDA-MB-231 at 72 h was also calculated, respectively, using SPSS software. **c** The synergistic effects of BA with taoxl was determined using the CCK-8 assays after 48 h treatment. The combination index (CI) for each data point was calculated with the CalcuSyn software for combined effect analysis. CI values of 0.9–1.1 is defined as additive, 0.3–0.9 as synergistic, and < 0.3 as strongly synergistic, whereas values > 1.1 is considered as antagonistic. Non-cytotoxicity doses of BA (0–8 μM) produced synergistic inhibitory effects with taxol (0–24 nM) on both breast cancer cells. **d** MCF-7 and MDA-MB-231 cells were treated with 8 μM BA and/or 4 nM taxol and subjected to colony formation assay. BA and taxol co-treatment resulted in a significant reduction on colony growth (All values represented as mean ± SD, *n* = 6, *P < 0.05)

To assess the direct cytotoxicity of BA, breast cancer cell lines, MCF-7, and MDA-MB-231 were treated with BA for 12, 24, 48, and 72 h and analyzed with CCK-8 proliferation assay. We found that BA produced dose-dependent cytotoxicity effects on both cell lines. Notably, inhibitory effect of BA on MCF-7 cells increased with time and peaked at 48 h after administration, whereas the peak value was 72 h for MDA-MB-231 cells (Fig. 1a). Meanwhile, minimal inhibitory effect was observed on the normal mammary epithelial cell line MCF-10A, indicating that BA might be a highly selective killing agent toward breast cancer cells (Fig. 1a). The half inhibitory concentration (IC_50_) of BA was 18.411 and 20.465 μM for MCF-7 and MDA-MB-231, respectively (Fig. 1b). In order to study the synergistic effects of BA with taxol, we optimized BA concentration to avoid its direct cytotoxicity effects on breast cancer cells. The combination index (CI) was analyzed by CalcuSyn software (CI values between 0.3 and 0.9 were defined as synergistic, < 0.3 as strongly synergistic). Results showed that BA plays strong synergistic effects with taxol on MCF-7 cells, while exerting moderate synergistic effects on MDA-MB-231 cells (Fig. 1c). To evaluate the long-term inhibitory effects of the combination treatment, colony formation assay was carried out. We found that BA cooperated with taxol to reduce the clonogenic ability of both breast cancer cells at a non-toxic concentration (Fig. 1d), further confirming the chemosensitizing effects of BA in a long-term administration manner.

### BA increases chemo-susceptibility of taxol-resistant breast cancer cells

**Fig. 2:**
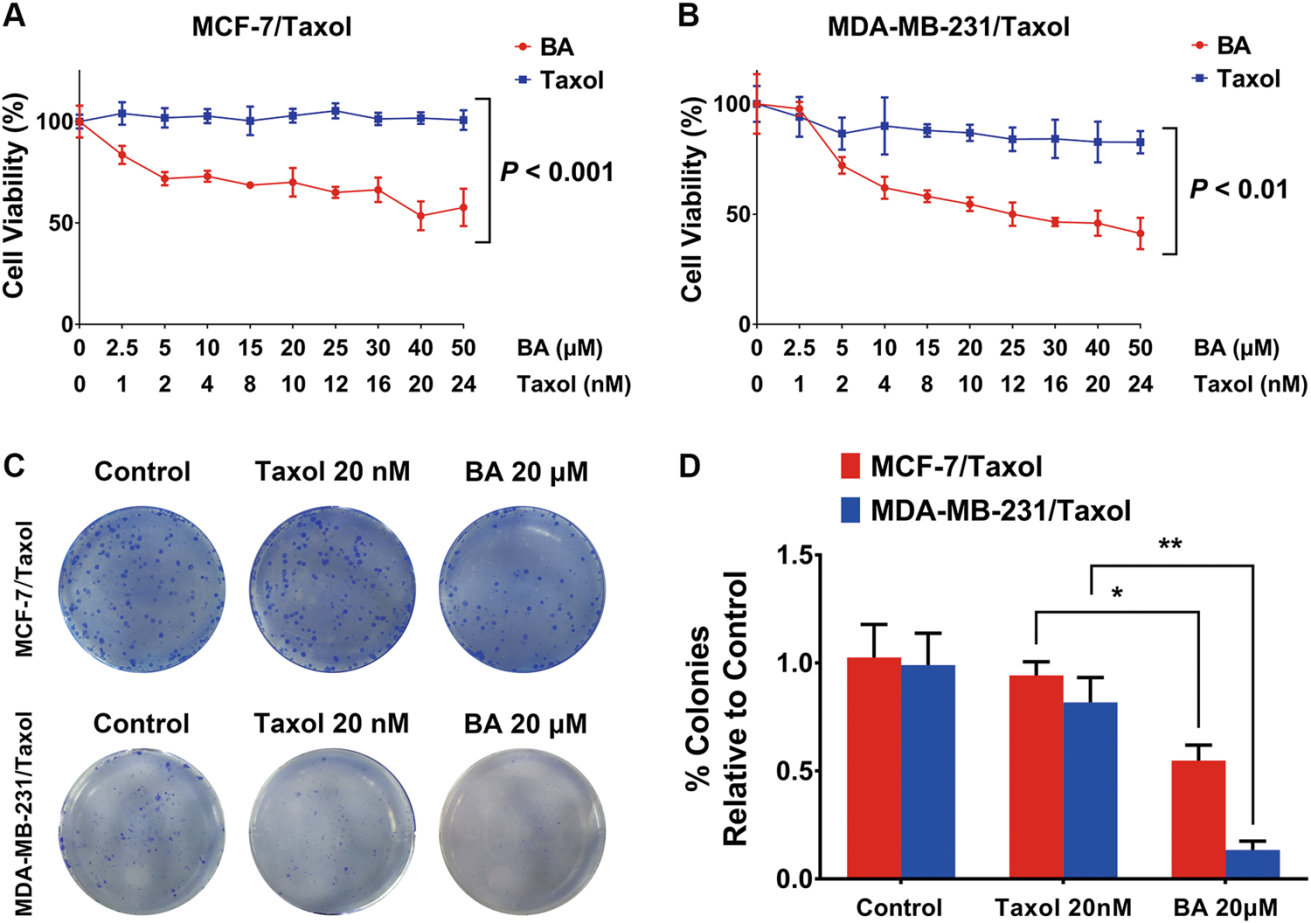
BA chemosenzitizes taxol-resistant breast cancer cells. **a**, **b** Taxol-resistant breast cancer cells MDA-MB-231 and MCF-7 were established by long-term drug co-culture system. BA still displayed significant inhibitory effects on MCF-7/taxol (*P* < 0.001) and MDA-MB-231/taxol (*P* < 0.01) cells. **c**, **d** BA restricted colony formation in both taxol-resistant cell lines, colonies percentage relative to control groups were statistically calculated (all values represented as mean ± SD, *n* = 6, *P < 0.05)

As BA increased chemosensitivity of breast cancer cells to taxol at non-toxic concentrations, we further tested the proliferation inhibition effects of BA on taxol-resistant breast cancer cell lines MCF-7/taxol and MDA-MB-231/taxol. As expected, taxol was virtually ineffective against both resistant cell lines. However, BA maintained inhibitory activity against proliferation of resistant cells within the same dosage range (Fig. 2a, b). Similarly, although taxol was not capable of restraining clonogenic cell survival at 20 nM in both resistant cell lines, BA was still capable of inhibiting their colony formation ability (Fig. 2c, d). These results further demonstrated the chemosensitizing effects of BA on drug-resistant breast cancer cells.

### BA enhances apoptosis induction and cell cycle arrest effects of taxol

**Fig. 3:**
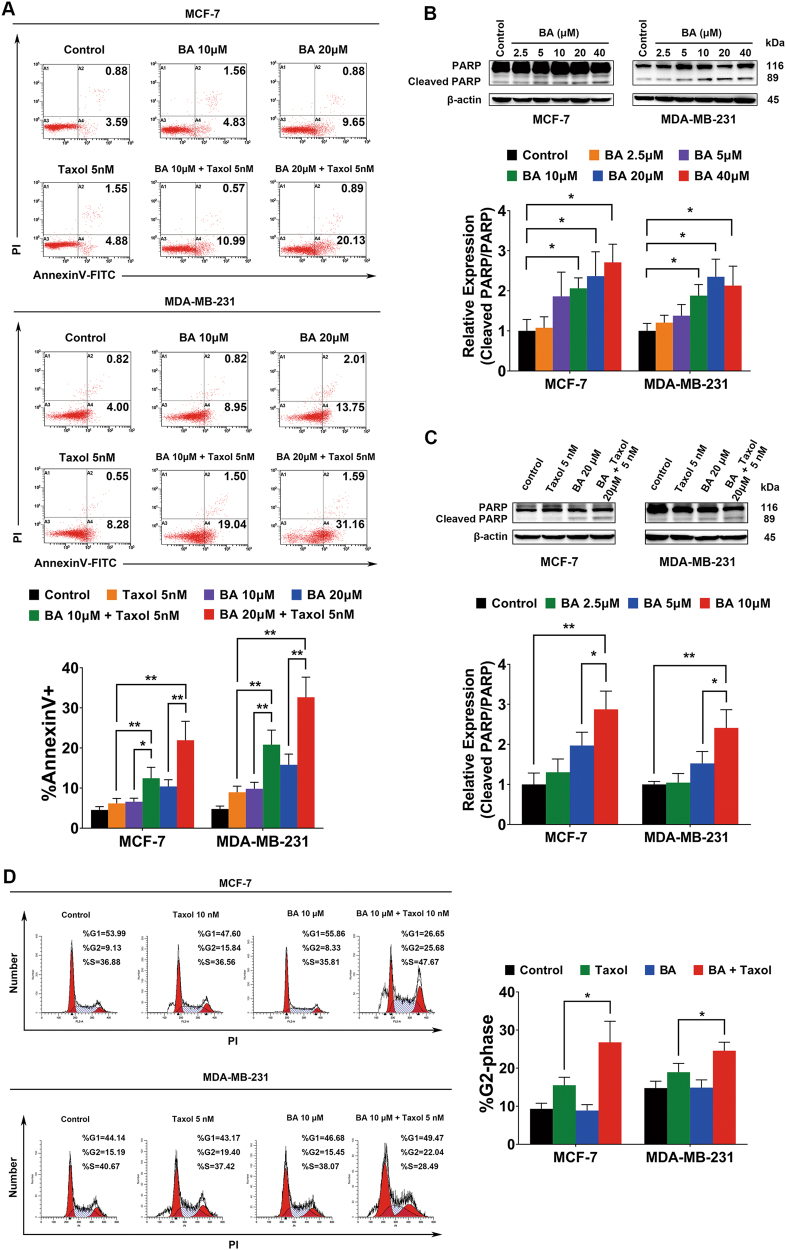
BA synergistically interacts with taxol to enhance breast cancer cell apoptosis and cell cycle arrest. **a** MCF-7 and MDA-MB-231 cells were treated with BA or taxol for 48 h and subjected to Annexin V-FITC/PI staining assay. BA displayed apoptosis induction effects on both breast cancer cells dose-dependently. Meanwhile, BA was also found to increase taxol-induced apoptosis in a dose-dependent manner. **b** The levels of PARP and cleaved PARP of breast cancer cells following BA treatment were determined by western blot analysis. The results showed that BA could dose-dependently increase cleaved PARP from 2.5 to 40 μM at 24 h. **c** BA cooperated with taxol to increase cleaved PARP expression, which was consistent with Annexin V-FITC/PI staining assay. **d** Cell cycle analysis revealed that BA synergistically interacted with taxol to arrest G2/M checkpoint. (all values represented as mean ± SD, *n* = 3, *P < 0.05, **P < 0.01)

We next investigated whether BA could synergistically aggravate apoptosis and cell cycle arrest effects induced by taxol. Treatment of breast cancer cells with a low dosage of taxol alone induced minimal apoptosis. However, when BA was administrated simultaneously with taxol, the apoptosis ratio was increased dose-dependently as determined by Annexin V-fluorescein isothiocyanate (FITC)/propidium iodide (PI) analysis (Fig. 3a). Meanwhile, western blotting results also revealed that BA efficiently induced cleavage of PARP in both MCF-7 and MDA-MB-231 cells in a dose-dependent manner (Fig. 3b). In addition, in line with the flow cytometry analysis, combined BA and taxol treatment increased cleaved PARP compared to either BA or taxol alone (Fig. 3c). As taxol is known to induce G2/M checkpoint arrest, we extended our studies to cell cycle analysis. Interestingly, although BA alone had little effects on cell cycle distribution at 10 μM, it significantly facilitated G2/M arrest induced by taxol (Fig. 3d). These results indicated that BA might enhance breast cancer chemosensitivity via apoptosis induction and cell cycle arrest.

### Identification of GRP78 as the direct interacting protein target of BA by DARTS

**Fig. 4:**
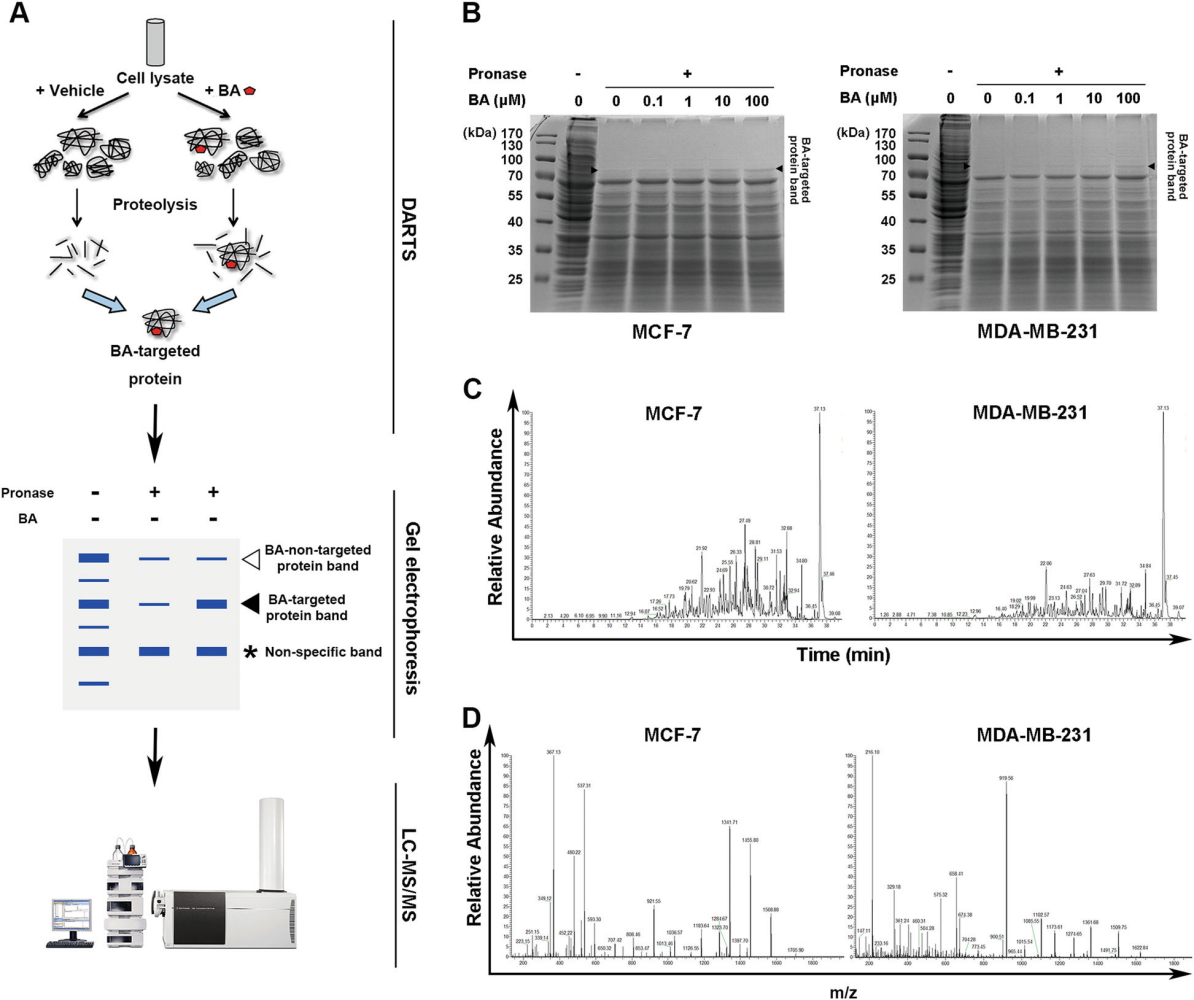
Identification of GRP78 as the direct target of BA in breast cancer cells by DARTS technique. **a** Schematic diagram of DARTS technique. **b** MCF-7 and MDA-MB-231 were treated with BA from 0.1–100 μM for 3 h. The cell lysates were proteolyzed with 0.05 mg/ml pronase for 30 min at 4 °C, followed by SDS-PAGE analysis and Coomassie blue staining. The black arrow indicates a protective band around 70 kDa. **c** Mass spectroscopy analysis of the BA-targeted protein was performed, and the first order mass spectrograms were shown. **d** The most probable target protein of BA was identified as GRP78. The representative peptide mass fingerprints of GRP78 were presented

Based on the above results, it was necessary to elucidate the precise molecular target of BA responsible for its chemosensitizing effects. DARTS technique is a well validated strategy to determine the direct molecular target of phytochemicals based on proteolysis protection after chemical-target binding (Fig. 4a). By utilizing this strategy, MCF-7 and MDA-MB-231 cells were treated with BA at 0.1–100 μM doses for 3 h followed by pronase-mediated proteolytic digestion. The protein lysates were separated and stained with Coomassie blue. DARTS revealed a strong protected band ~ 70 kDa in the BA-treated lanes, which was present in a dose-dependent manner (Fig. 4b). The protected bands were then analyzed by LC/MS and identified as GRP78 (Fig. 4c, d). These results revealed that BA might enhance breast cancer chemosensitivity via directly interacting with GRP78.

### BA enhances apoptosis induction primarily due to activation of ER stress

**Fig. 5:**
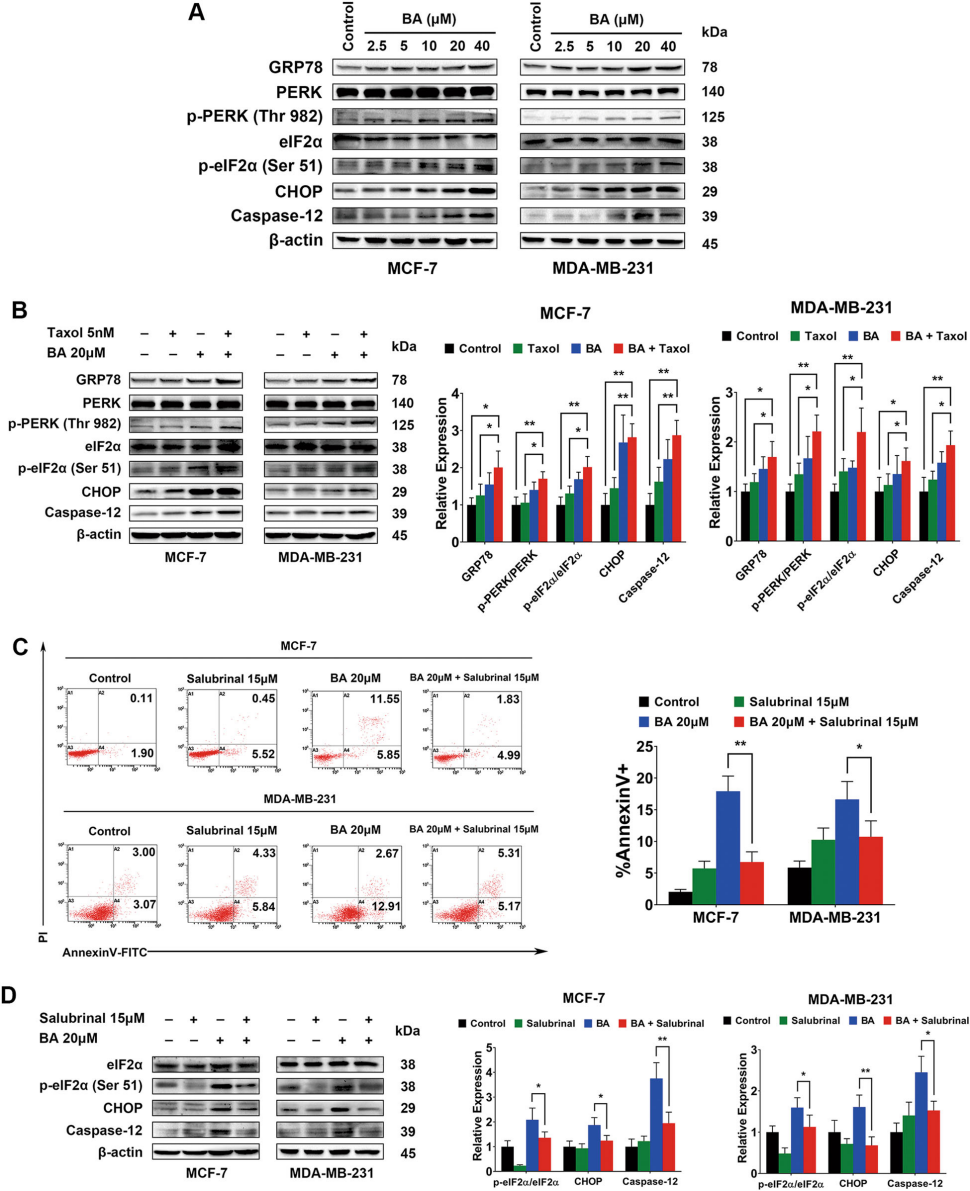
BA triggers breast cancer cells apoptosis *via* ER stress-mediated pathway. **a** MCF-7 and MDA-MB-231 cells were treated with the indicated concentrations of BA for 24 h, and the protein levels of ER stress-associated signals were stimulated by BA in a dose-dependent manner, including GRP78, p-PERK/PERK, p-eIF2α/eIF2α, CHOP, and caspase-12. **b** MCF-7 and MDA-MB-231 cells were treated with BA alone or in combination with taxol for 24 h, the expression levels of GRP78, p-PERK/PERK, p-eIF2α/eIF2α, CHOP, and caspase-12 were also significantly upregulated following drug administration, especially in the co-treatment group, indicating the ER stress-mediated apoptosis pathway was aggravatedly activated by drug combination. **c** MCF-7 and MDA-MB-231 cells were treated with ER stress inhibitor salubrinal or BA for 48 h. Flow cytometry analysis revealed that salubrinal significantly attenuates the apoptosis induction effects of BA, indicating ER stress signaling plays a critical role in the anticancer activities of BA. **d** Protein lysates of both breast cancer cells were also subjected to western blot analysis. Salubrinal inhibited eIF2α phosphorylation and reduced levels of CHOP and Caspase-12 induced by BA, further validating the critical contribution of ER stress underlying the activities of BA (all values represented as mean ± SD, *n* = 3, *P < 0.05, **P < 0.01)

As GRP78 plays a critical role in mediating ER stress, we therefore determined the expressions of proteins involved in the ER stress modulation pathway following BA administration. We found that GRP78 expression was significantly increased by BA in a dose-dependent manner. In addition, the GRP78 downstream signaling including p-PERK and p-eIF2α were also increased simultaneously. Moreover, CHOP and Caspase-12, which play a central role in ER stress-mediated apoptosis, were activated upon BA treatment (Fig. 5a). Therefore, we next explored whether BA could increase chemosensitivity of taxol via GRP78-mediated apoptosis. The results showed that 5 nM taxol only induced moderate elevation of GRP78, p-PERK, and p-eIF2α. However, their expressions were significantly increased following BA co-treatment, accompanied by an increased level of CHOP and Caspase-12, implying that ER stress apoptotic pathway was involved in the chemosensitizing effects of BA (Fig. 5b). To demonstrate the significant role of ER stress in mediating BA bioactivity, we administrated BA and salubrinal together, which is an ER stress inhibitor by blocking eIF2α activity^27,28^. Annexin V-FITC/PI staining assay demonstrated that salubrinal significantly suppressed the apoptosis-inducing effects of BA, indicating that ER stress-mediated apoptosis induction might be mainly responsible for BA’s chemosensitizing effects (Fig. 5c). Moreover, we also examined the expressions of CHOP and Caspase-12 with or without salubrinal treatment. Similarly, we found that salubrinal administration significantly inhibited the increased CHOP and Caspase-12 induced by BA (Fig. 5d). All these results indicated that BA could increase breast cancer chemosensitivity via ER stress-mediated apoptosis pathway (Fig. 5d). It is generally considered that ER, as a calcium reservoir, plays a key role in regulating transduction and amplification of apoptotic signals. Therefore, the intracellular calcium concentration ([Ca^2+^]_i_) was also detected after BA treatment. We found that BA significantly elevated intracellular free calcium concentration (Supplementary Figure 1). As ΔΨm might be damaged due to the increased calcium exposure, it was also recorded that the ΔΨm was disrupted following BA administration in breast cancer cells (Supplementary Figure 2). Meanwhile, the increased Cytochrome c and Bax, and the downregulation of Bcl-2 (Supplementary Figure 3) further demonstrated that the mitochondrial pathway apoptosis was also activated by BA treatment.

### BA triggers ER stress-mediated apoptosis pathway via targeting GRP78

**Fig. 6:**
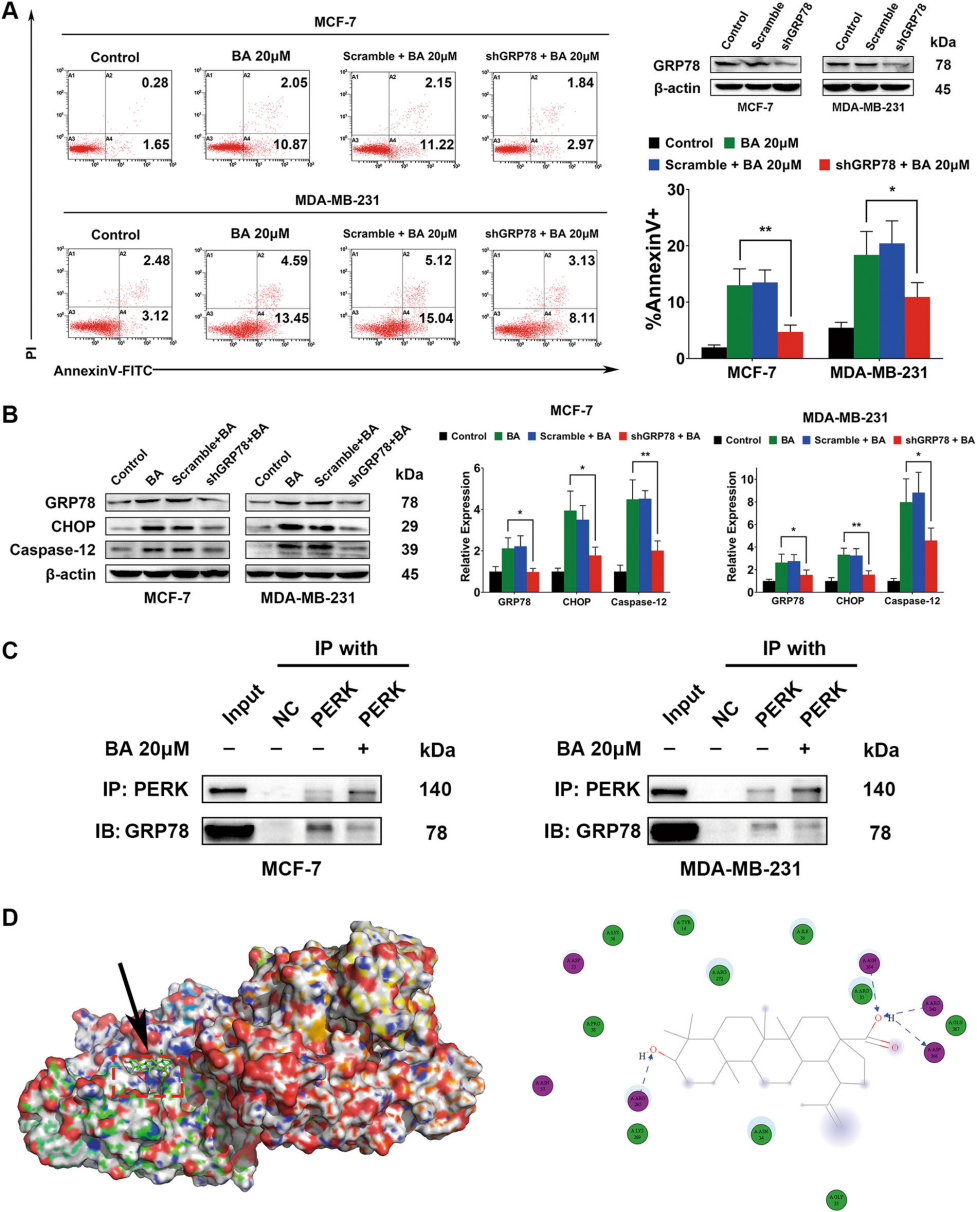
BA triggers ER stress-mediated apoptosis via targeting GRP78. **a** MCF-7 and MDA-MB-231 cells were transfected with shRNA plasmid against GRP78 (shGRP78) or the scramble plasmid (Scramble) and validated by immunoblotting analysis. Annexin V-FITC/PI flow cytometry assay revealed that GRP78 silencing inhibited BA-induced apoptosis in both breast cancer cells. **b** GRP78 silencing also downregulated the expressions of CHOP and Caspase-12 induced by BA. **c** Coimmunoprecipitation analysis validated that BA administration could interrupt the interaction between GRP78 and PERK. **d** Molecular docking analysis revealed that BA could stably dock into the ATPase-binding site of GRP78 and form hydrogen bonds with active residues including ARG265, ASN364, ARG342, and ASP366 (all values represented as mean ± SD, *n* = 3, *P < 0.05, **P < 0.01)

Although we demonstrated the effects of BA on GRP78 and ER stress-mediated apoptosis, the central role of GRP78 in mediating the bioactivity of BA remained unclear. Therefore, GRP78 was further silenced in both MCF-7 and MDA-MB-231 cells to confirm whether GRP78 knockdown would inhibit the apoptosis-inducing effects of BA. The GRP78 level was significantly decreased following its small hairpin RNA (shRNA) transfection (Fig. 6a). Compared with cells treated with BA only, GRP78 knockdown led to a significant reduction of apoptosis ratio induced by BA (Fig. 6a). Meanwhile, the expressions of CHOP and Caspase-12 upregulated by BA were also decreased following GRP78 knockdown (Fig. 6b). To provide more convincing evidence unraveling the interaction between GRP78 and BA, we performed coimmunoprecipitation between GRP78 and PERK. The results showed that their interaction was interrupted following BA treatment (Fig. 6c). PERK disassociation from GRP78 subsequently initiated ER stress-mediated apoptosis. These findings suggested that BA triggered ER stress-mediated apoptosis via interrupting the binding between GRP78 and PERK. To further explore the binding mode of BA with GRP78 at the molecular level, we conducted a molecular docking analysis using a homology model 3LDP at the catalytic site of GRP78 crystal structure. Results revealed that BA could be stably docked into the ATPase domain of GRP78 with binding energy of 6.91 kcal/mol. In particular, BA could form stable hydrogen bonds with four active residues including ARG265, ASN364, ARG342, and ASP366 (Fig. 6d), further providing evidence that BA interrupts the interaction between GRP78 and PERK.

### BA enhances taxol chemosensitivity in breast cancer xenografts

**Fig. 7:**
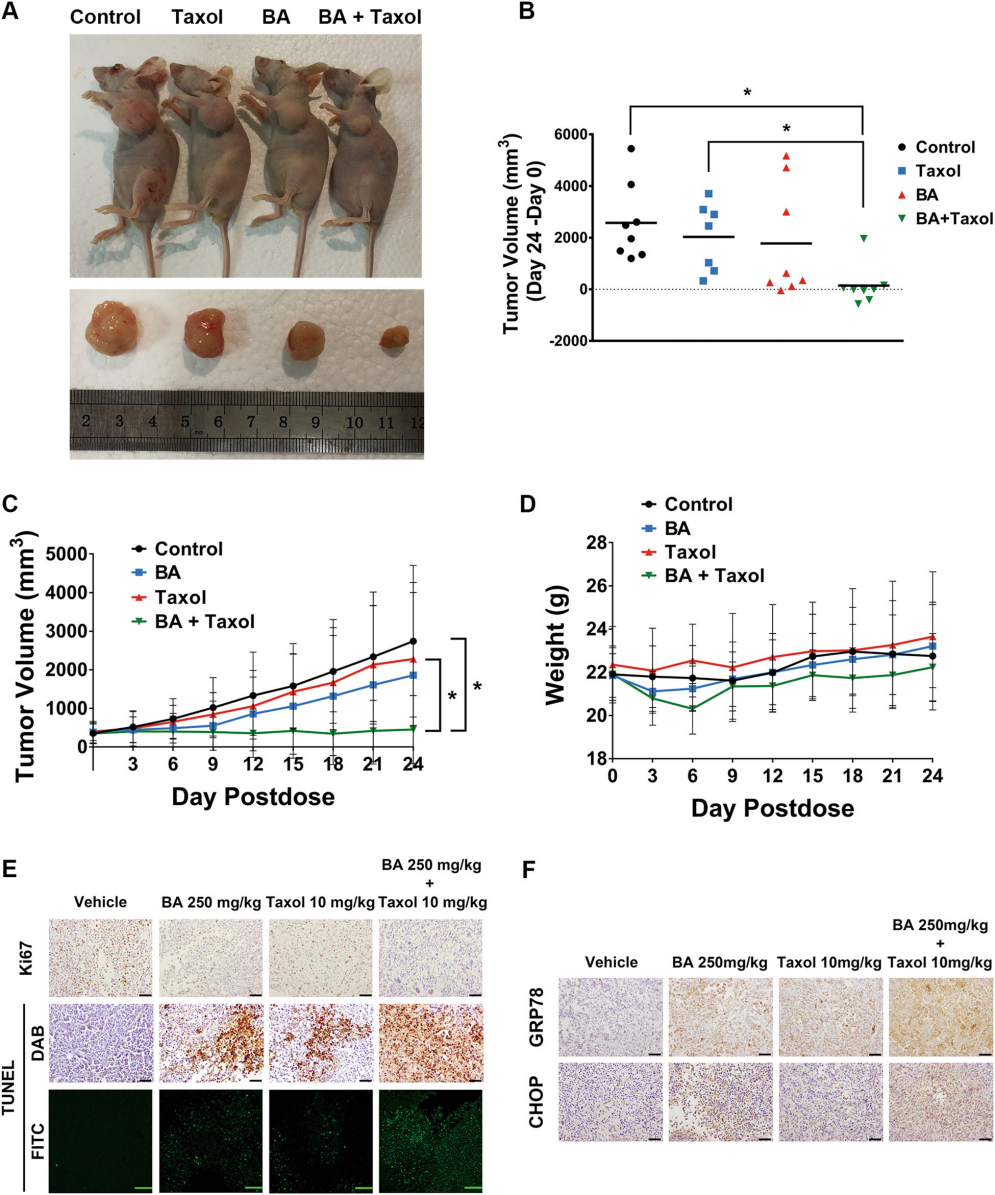
In vivo chemosensitizing effects of BA on breast cancer xenografts. **a** Representative tumor image of each treatment group. **b**, **c** BA significantly inhibits breast cancer growth in vivo, and could synergistically enhance taxol therapeutic efficacy (all values represented as the mean ± SD, *n* = 8, *P < 0.05). **d** BA and low-dose taxol brought little influence on mice body weight, but their co-treatment led to body weight loss. **e** TUNEL assay revealed that BA synergistically interacted with taxol to induce apoptotic events in vivo, accompanied with Ki67 reduction. **f** Immunohistochemistry analysis showed that BA could enhance GRP78 and CHOP expression in vivo. Meanwhile, BA plus with taxol co-treatment could significantly induce more accumulative GRP78 and CHOP expression in tumor tissue. All scale bars indicate 100 μm

Based on in vitro results, we sought to determine the synergistic effects between BA and taxol in vivo. Breast cancer xenografts were established by subcutaneously injecting MDA-MB-231 cells into the mammary glands of Balb/c-nu/nu mice. Treatment with a 10 mg/kg of taxol alone only had slight antitumor effects. Nevertheless, combined application of BA and taxol significantly inhibited tumor growth (Fig. 7a–c). Meanwhile, BA or taxol did not result in significant weight loss throughout the experiment duration. In addition, BA did not induce any morphological variations in the liver, heart, spleen, lung, and kidney (data not shown). These results were consistent with the low toxicity of BA on normal cells observed in vitro. However, when BA and taxol were administrated together, body weight loss was observed, highlighting the increased toxic effects when applied simultaneously. Meanwhile, TUNEL assays revealed that the combined treatment of BA with taxol induced an enhanced apoptosis ratio in tumor tissue (Fig. 7d). Correspondingly, Ki67 expression was also sharply decreased when both agents were co-administrated (Fig. 7e). As in vitro studies suggested the critical role of ER stress-induced apoptosis in mediating chemosensitizing effects of BA, the expression of CHOP and GRP78 was also detected. Consistent with in vitro results, immunohistochemistry assay showed that BA could significantly upregulate the expression of GRP78. Moreover, combined application of BA and taxol resulted in a significantly higher level of CHOP than treated alone, further confirming our findings in vitro (Fig. 7f).

## Discussion

Development of chemoresistance has long been a major obstacle in cancer therapy and accounts for over 90% cancer-related deaths. Although research endeavor and efforts have been dedicated to explore the molecular targets involved, the underlying mechanisms of chemoresistance have largely remained unknown. Recent studies highlight the critical role of stress-related machinery in protecting cells away from harmful damage induced by toxic drugs. Stress-related signaling, such as enhanced expressions of ROS scavenging enzymes, autophagy induction, and glycolysis activation, have been highly implicated in the process of acquired drug resistance^29^. Meanwhile, a number of low-toxic phytochemicals have displayed encouraging effects on chemosensitivity enhancement through stress modulation. For example, curcumin was found to enhance irinotecan efficiency on colorectal cancer through ROS generation and ER stress activation^30^. Resveratrol was capable of inducing COX-2-dependent apoptosis in human ovarian cancer cells^31^. Seeking natural stress-regulated molecules seems to be a promising approach to improve cancer chemosensitivity with reasonable safety. In the current study, we also showed that BA could chemosensitize breast cancer through triggering stress overload both in vitro and in vivo.

BA has displayed anticancer activities against multiple malignancies including breast, liver, prostate, colorectal, cervical, and pancreatic cancers^24^. Several targets were reported to be involved in the bioactivities of BA, such as NF-κB, P53, PI3K, ERBB2, STAT3, ERα, and HIF-1α^32^. Recent studies suggested that BA mediated antitumor activity via degrading the transcription factor Sp1, and cDNA array identified that the SP1 downstream gene Lamin B1 critically contributes to the anticancer activities of BA^33^. However, both in vitro cell model and genetic mouse xenografts demonstrated that Sp1 inhibition is dispensable for cell growth and differentiation^34^, indicating that molecular targets other than Sp1 might be responsible for the selective cancer killing effects of BA. Similar to previous reports, our study also found that BA could selectively inhibit breast cancer cell growth^22^ and cooperated with anticancer drugs to induce apoptosis in several tumor cell lines^35^. We initially identified the ER stress modulator protein GRP78 as the direct target of BA through DARTS strategy. DARTS is a well-established universal applicable drug-target identification approach based on a reduction in the protease susceptibility of the target protein upon drug binding. Compared with other affinity-based target identification system, the key advantage of DARTS is that it does not require labeled ligands and large amounts of pure protein. Moreover, DARTS is completely independent of any intracellular pharmacological action of the drug, making it useful for any small molecule of interest^36–38^. Based on our DARTS results, we further found that BA could directly interrupt the interaction between GRP78 and PERK, which triggers downstream apoptosis cascade. Molecular docking analysis revealed that BA could competitively bind with the ATPase domain of GRP78, which might account for the disassociation of GRP78 and PERK. Thus, we have uncovered a novel target underlying anticancer bioactivities of BA, and our study potentially provides an optimizing strategy for designing BA-based therapeutics.

**Fig. 8:**
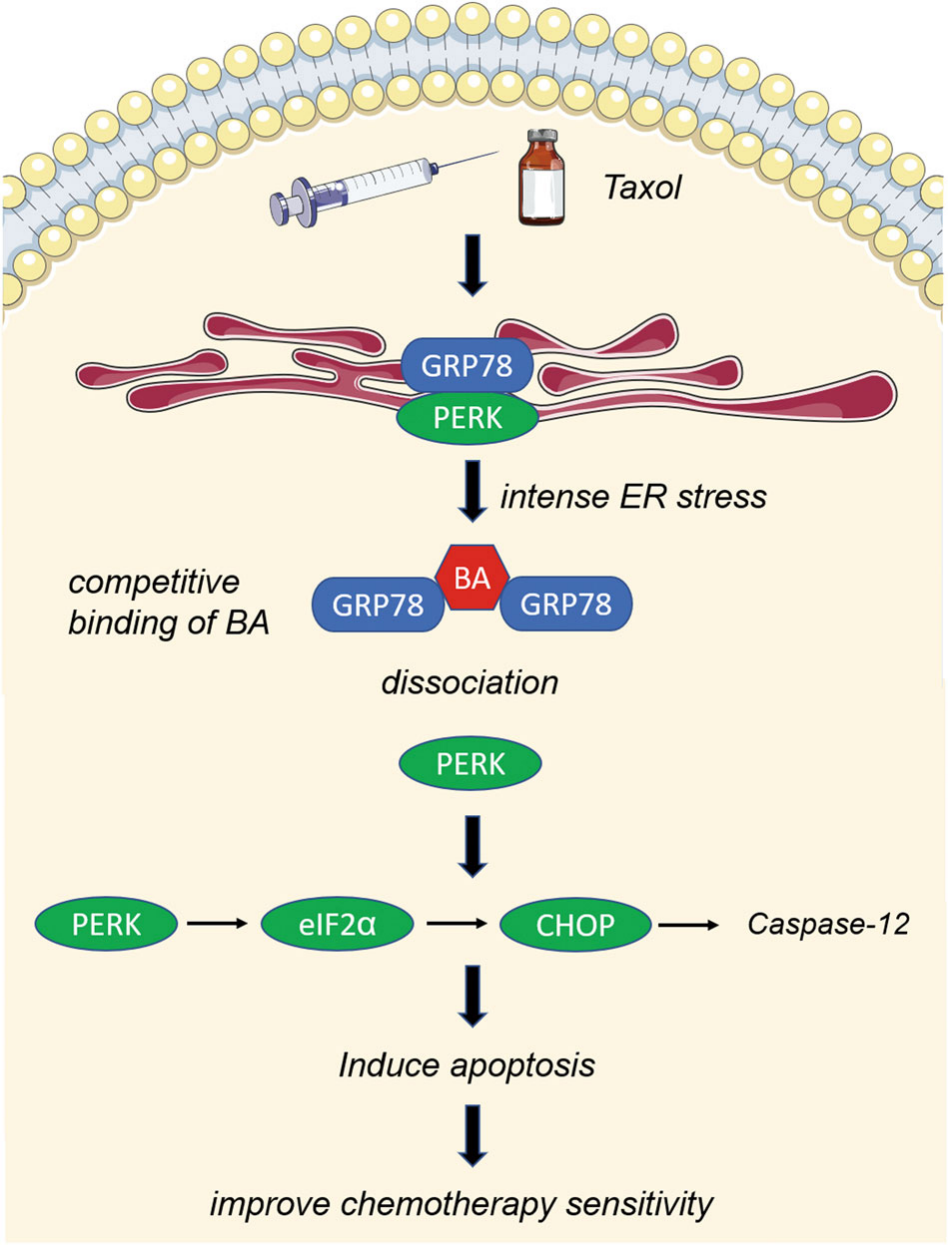
Diagram illustrating the involvement of GRP78 in mediating BA-induced chemosensitization against breast cancer

GRP78, also known as BiP, is a major UPR regulator, which plays important roles in protein folding, degrading misfolded proteins, and Ca^2+^ binding. As a stress-response protein, GRP78 overexpression has been observed in a number of malignancies, and GRP78 upregulation was found to promote cancer angiogenesis, drug resistance, and metastasis^9,39^. Upon chemotherapy, GRP78 was reported to confer resistance to 5-FU by directing interacting with c-Src kinase^40^. Similarly, GRP78 could also activate EGFR to increase hepatocellular carcinoma resistance to sorafenib^41^. In the present study, we also found that GRP78 expression was significantly upregulated following taxol or BA treatment, which is consistent with previous reports as stated above. However, overexpressed GRP78 expression was accompanied by increased apoptosis ratio and cleaved PARP expression in BA alone and combination groups, indicating that pharmacological-induced GRP78 upregulation also activates the ER stress-mediated apoptotic pathway. Under persistent or intense stress stimulus, GRP78 is disassociated from stress sensor PERK, which in turn activates the PERK/eIF2α/CHOP pathway and initiates the apoptotic process by upregulating Caspase-12 (Fig. 8). These findings suggest that BA might aggravate the stressful microenvironment induced by taxol, and consequently result in GRP78 hyperactivation. Meanwhile, it is likely that GRP78 might be a double-edged sword during cancer stress modulation. Transient activation of GRP78 could improve cell survival by degrading the damaged proteins induced by harmful stress, whereas persistent GRP78 activation would lead to apoptosis induction via PERK/eIF2α/CHOP pathway.

Growing evidence also suggests that agents affecting ER stress could be exploited as promising anticancer drug candidates. Proteasome degradation plays a critical role in alleviating ER stress, and proteasome inhibition will undoubtedly increase the protein burden on the already challenged ER and subsequently tilt the balance toward apoptosis^42,43^. Proteasome inhibitor Bortezomib has reached successful clinical trials and exhibited most effective anticancer activities against hematopoietic tumors^44^. Meanwhile, combination of Bortezomib and cisplatin resulted in a severe sensitization of pancreatic cancer cells to apoptosis^45^. Brefeldin A, which causes an accumulation of trapped secretory proteins in the ER, could also induce apoptosis in several malignancies such as leukemia, colon cancer, prostatic adenocarcinoma, and melanoma^46^. Thapsigargin, a classic ER stress inducer, increases the cell killing potency of taxol by 10 to 12-fold in human prostate cancer PC-3 cells^47^. Therefore, provoking ER overload stress is emerging as an interesting therapeutic strategy. However, special attention must be paid to the safety issue during strategy design and a highly specific molecular target is the most critical factor. Since GRP78 was reported to be highly elevated in multiple malignancies and has restricted expression at the plasma membrane^48^, GRP78 is now becoming an attractive and ideal target for the development of new therapeutic tactics. Our study provides a new candidate drug for GRP78 targeting, which had minimal influence on the normal cell viability. These findings further implicate the feasibility of GRP78 targeting strategy for cancer chemosensitizing therapy.

Taken together, our study demonstrated that BA chemosensitized breast cancer through directly targeting GRP78 to trigger ER stress-mediated apoptotic pathway, thus uncovering a novel mechanism underlying the potent antitumor effects of BA in breast cancer. Meanwhile, GRP78-based chemosensitizing strategy is also highlighted as a novel approach to improve cancer prognosis. The role and regulation mechanisms of GRP78 and its natural targeting agent BA warrant more studies to ensure an eventual translation into clinical application.

## Materials and methods

### Cell culture and selection of taxol-resistant breast cancer cells

The human breast cancer cell lines MCF-7, MDA-MB-231 were obtained from the American Type Culture Collection (ATCC). The cells were cultivated in medium supplemented with 10% fetal bovine serum and 1% penicillin and streptomycin (Gibco Life Technologies, Lofer, Austria) at 37 °C in a humidified incubator with or without 5% CO_2_. MCF-7/taxol and MDA-MB-231/taxol cells are taxol-resistant clones selected from parental MCF-7 and MDA-MB-231 cells. In brief, parental cells were treated with increasing taxol (Bristol-Myers Squibb) concentrations in culture medium for selection. After 6 months, resistant cell clone was obtained and used for subsequent experiments. The normal mammary epithelial cells MCF-10A were also purchased from ATCC and cultured in DMEM/F12 supplemented with 5% horse serum, 1% penicillin and streptomycin (Gibco Life Technologies, Lofer, Austria), 20 ng/ml recombinant human epidermal growth factor, 0.5 μg/ml hydrocortisone, 100 ng/ml cholera toxin and 10 μg/ml insulin (Sigma-Aldrich, Shanghai, China).

### Cell viability and combined effect analysis

CCK-8 assays were performed to assess the cell viability. In brief, the MCF-7, MDA-MB-231, MCF-7/taxol, MDA-MB-231/taxol, and MCF-10A cells were seeded onto 96-well plates at a density of 3 × 10^3^ cells per well. After cells attachment, serial concentration gradients of BA (Xi’an Natural Field Bio-Technique Co., Ltd, Xi’an, China) or taxol were added to the wells, with six repeats for each concentration. Cell viability was then detected using CCK-8 agents according to the manufacturer’s instructions (KeyGEN BioTECH, Jiangsu, China). A triplicate independent experiment was performed. The combination effects and a potential synergic interaction were evaluated from quantitative analysis of dose-effect relationship. CI was calculated using the CalcuSyn software. The analysis generally defines CI values of 0.9–1.1 as additive, 0.3–0.9 as synergistic, and <0.3 as strongly synergistic, whereas values >1.1 is considered as antagonistic. To determine the long-term inhibitory effects, a colony formation assay was conducted. MCF-7, MDA-MB-231, and MCF-7/taxol, MDA-MB-231/taxol cells were seeded onto six-well plates at a density of 1000 cells per well. After cell attachment, BA or taxol was added to the wells alone or in combination. The cells were then cultured with fresh medium. After 2 weeks, the resultant colonies were fixed with 4% paraformaldehyde and stained with Coomassie blue.

### Cell apoptosis analysis

Cell apoptosis was evaluated by flow cytometric analysis of Annexin V-FITC/PI staining. In brief, cells were plated with an initial cell number of 2 × 10^5^/ml in six-well plates. The exponentially growing cells were exposed to indicated doses of the BA and taxol individually or in a combination for 48 h. Annexin V-FITC/PI staining was performed according to the manufacturer’s instruction (Multi Sciences, Hangzhou, China). Cells positive for Annexin V-FITC and negative for PI are considered as early-stage apoptosis subpopulation. Cells positive for both Annexin V-FITC and PI are calculated as late-stage apoptosis subpopulation. A triplicate independent experiment was performed.

### Cell cycle analysis

Flow cytometric analysis was performed using PI/RNase staining according to standard procedures. After treatment with BA and/or taxol for 24 h, cells were collected by centrifugation, fixed, and permeabilized in 70% cold ethanol overnight at −20 °C. Samples were washed with cold PBS and incubated with the PI (5 mg/ml) and RNase (1 mg/ml) (Sigma-Aldrich, Shanghai, China) at room temperature for 30 min before analysis. Histograms plotted were then analyzed with Multicycle software. A triplicate independent experiment was performed.

### Western blotting analysis

Cells were lysed in radioimmunoprecipitation assay buffer (Sigma-Aldrich, Shanghai, China) containing a protease and phosphatase inhibitor mixture (Roche Diagnostics, Shanghai, China). The protein concentration was measured with the bicinchoninic acid assay (Sigma-Aldrich, Shanghai, China). Quantified protein lysates (50 µg) were resolved on sodium dodecyl sulfate polyacrylamide gel electrophoresis (SDS-PAGE) gel, transferred onto PVDF membrane (Millipore, Billerica, MA), and immunoblotted with antibodies including Cytochrome c antibody (4272, Cell Signaling Technology, Danvers, MA, USA), Bcl-2 antibody (12789-1-AP, Proteintech, Rosemont, IL, USA), Bax antibody (A7626, ABclonal Technology Cambridge, Boston, USA), PARP antibody (9532, Cell Signaling Technology, Danvers, MA, USA), β-actin antibody (4970, Cell Signaling Technology, Danvers, MA, USA), GRP78 antibody (sc-376768, Santa Cruz Biotechnology, Santa Cruz, CA, USA), PERK antibody (5683, Cell Signaling Technology, Danvers, MA, USA), Phospho-PERK antibody (DF7576, Affinity Biosciences, Cincinnati, OH, USA), eIF2α antibody (AF6087, Affinity Biosciences, Cincinnati, OH, USA), Phospho-eIF2α antibody (AF3087, Affinity Biosciences, Cincinnati, OH, USA), CHOP antibody (15204-1-AP, Proteintech, Rosemont, IL, USA) and Caspase-12 antibody (55238-1-AP, Proteintech, Rosemont, IL, USA) at 4 °C overnight. After three washes using Tris-buffered saline with 0.05% Tween-20, the membrane was incubated with secondary anti-rabbit or anti-mouse antibodies (Sigma-Aldrich, Shanghai, China) for 1 h at room temperature. The signals were visualized using the ECL Advance reagent (Tanon Science & Technology, Shanghai, China) and quantified using the ImageLab software.

### Mitochondrial membrane potential (ΔΨm) evaluation

Cells were treated with 20 and 40 μM BA or the vehicle control for 24 h, followed by incubation with JC-1 fluorescence probe (Beyotime Biotechnology, Shanghai, China) at 37 °C in a humidified incubator with 5% CO_2_ for 20 min. Then, cells were washed twice with staining buffer, and the pellet was suspended and analyzed using Cytomics^TM^ FC 500 (Beckman Coulter, CA, USA). The emission of JC-1 monomers was detected at 530 nm, and JC-1 aggregates were detected at 590 nm. A loss of aggregates and an increase in monomers signal the disruption of the mitochondrial transmembrane potential (ΔΨm).

### Detection of free [Ca^2+^]_i_

MCF-7 and MDA-MB-231 cells were treated with 20 and 40 μM BA for 24 h, then were assessed for [Ca^2+^]_i_. Cells were incubated with 2.5 μM calcium fluorescence probe Fluo-4/AM (Beyotime Biotechnology, Shanghai, China) dissolved in dimethyl sulfoxide (DMSO) at 37 °C in a humidified incubator with 5% CO_2_ for 30 min. The fluorescence was recorded by a laser scanning confocal microscopy LSM 710 (Zeiss, Oberkochen, Germany).

### DARTS

The protein target of BA was identified by DARTS strategy according to the protocol by Lomenick et al.^36,37^. MCF-7 and MDA-MB-231 were treated either with varying concentrations of BA (0.1–100 μM) in DMSO or with DMSO alone (control). After 3 h of treatment, cells were collected and lysed in lysis buffer with protease and phosphatase inhibitors. Protein concentrations were determined by the bicinchoninic acid protein assay kit (Thermo Fisher Scientific) to ensure an equal amount of protein lysate per sample. For proteolysis, each cellular lysate sample was proteolyzed at 4 °C for 30 min with 0.05 mg/ml pronase (Roche Diagnostics, Indianapolis, USA). Then samples were loaded on SDS-PAGE and gels were stained with Coomassie blue. In the end, specific gel bands that were seen in treatment samples compared to controls were cut out and processed with trypsin digestion for mass spectrometry analysis.

### Molecular docking

The Autodock 4.2.6 was used in the molecular docking study. The chemical structure of BA was drawn by Chemoffice (CambridgeSoft, Cambridge, MA). The crystal structure of GRP78 was obtained from the Protein Data Bank (http://www.rcsb.org/pdb/) with the ID of 3LDP. The water molecules in GRP78 were removed. For docking purposes, the center of the binding site was determined by 3P1, an inhibitor of GRP78. The binding site was defined as a 30 × 30 × 30 Å cube box. The potentials to form hydrogen bonds within the active site were calculated. Docking with no output pose was considered a failure.

### Plasmids and shRNA transfection

shRNAs were purchased from GenePharma. Sequences of shGRP78 were as followings: GGTTACCCATGCAGTTGTTAC. Negative control cell lines were generated by infecting cells with scrambled plasmids. MCF-7 and MDA-MB-231 cells were transfected with plasmids using lipofectamine 2000 (Invitrogen, Carlsbad, CA, USA). In brief, cells were plated so they will be 70–90% confluent at the time of transfection. lipofectamine 2000 reagent and plasmid were diluted in Opti-MEM^®^ Medium and mixed at 1:1 ratio incubating for 5 min at room temperature. Then, plasmid DNA-lipid complexes were added to cells. After 48 h, the transfected cells were verified by western blot.

### Coimmunoprecipitation analysis

Immunoprecipitation assay was conducted by using the Pierce® Co-Immunoprecipitation Kit (no.26149, Thermo Fisher, Hudson, NH, USA) according to the manufacturer’s instructions. Primary antibodies for PERK (5683, Cell Signaling Technology, Danvers, MA, USA) and GRP78 (sc-376768, Santa Cruz Biotechnology, Santa Cruz, CA, USA) were used in this assay. PERK antibody was immobilized with AminoLink plus coupling resin first. MCF-7 and MDA-MB-231 cells were then lysed using IP lysis buffer. The pre-clear lysate was added to the control agarose resin to avoid nonspecific interactions with the resin matrix. Next, the coimmunoprecipitation process was performed by incubating treated lysate with PERK antibody immobilized coupling resin, the quenched antibody coupling resin as a negative control at the same time. Finally, the resin was heated at 100 °C for 5–10 min with SDS sample buffer to prepare SDS-PAGE analysis, and the target protein of GRP78 was detected by Western blot.

### Breast cancer xenografts in nude mice

All animals feeding and experimental treatment were authorized by institutional animal research ethics committee of Guangdong Provincial Hospital of Chinese Medicine. Five-week-old female Balb/c-nu/nu mice were obtained from the Animal Experimental Center of the Guangzhou University of Chinese Medicine. The mice were kept in specific pathogen free ventilation chambers under ambient temperature of 20–25 °C and 45–50% relative humidity. The rearing facility was maintained on a 12 h light–dark cycle. All mice were given habituation for 3 days before experiments with free access to sterilized food and water. Cells suspension of MDA-MB-231 was injected into mammary fat pads of each mouse (at the cell density of 5 × 10^6^ in 200 μl phosphate-buffered saline). When the tumor volume reached approximately 100 mm^3^, mice were randomly divided into control and various treatment groups (*n* = 8). Treatment groups are divided as follows: control group with vehicle; BA group (250 mg/kg); taxol group (10 mg/kg), and taxol plus BA group. BA and vehicle were given every 2 days by intraperitoneal administered, and taxol was given by intraperitoneal injection every 3 days. Mice were monitored for 24 days during treatment. Tumor volume was measured every 2 days and calculated with the formula ([width]^2^ × [length]/2). The body weight was also recorded throughout the whole experiment period.

### Immunohistochemistry

Tumor specimens were fixed in 10% neutral buffered formalin for 24 h, followed by standard tissue processing and embedding. Paraffin-embedded tumor sample sections were cut at 3 μm and dried overnight at 37 °C. The sections were then deparaffinized in xylene twice for 10 min each and rehydrated using a graded series of ethanol. Endogenous peroxidase was inactivated by incubating the sections in 3% hydrogen peroxide for 30 min at room temperature. Antigen retrieval was performed by heating the slides in the sodium-citrate buffer. The slides were then subjected to incubation with primary antibodies including Ki67 (9027, Cell Signaling Technology, Danvers, MA, USA), GRP78 (sc-376768, Santa Cruz Biotechnology, Santa Cruz, CA, USA), CHOP (15204-1-AP, Proteintech, Rosemont, IL, USA) at 4 °C overnight in a moist chamber. DAB detection system (ZSGB-BIO, Bejing, China) was applied as chromogenic agents according to the manufacturer’s instructions. Finally, sections were counterstained using Mayer’s hematoxylin, dehydrated, cleared, and mounted before the examination.

### TUNEL analysis

In situ cellular apoptosis of tumor tissues was evaluated by detection of fragmented DNA on deparaffinized formalin-fixed sections via TUNEL technique. In brief, paraffin-embedded tumor sections were deparaffinized and treated with proteinase K to strip proteins from the nuclei. Then the sections were incubated with terminal deoxynucleotidyl transferase at 37 °C for 1 h. Streptavidin-FITC was then added to the samples, followed by incubation in a humidified chamber for 30 min at 37 °C. The fluorescence intensity was visualized at an excitation wavelength of 450–500 nm and an emission wavelength of 515–565 nm. For TUNEL IHC assay, 50 μl of POD-conjugated anti-FITC was added and incubated with the tissue section for 30 min at 37 °C. 3, 5-Diaminobenzidine was then used to detect the sites of peroxidase binding, followed by counterstaining with hematoxylin.

### Statistical analysis

Data analysis was performed with Statistical Product and Service Solutions (SPSS) 19.0 software. The data were expressed as mean ± SD. Student’s *t* test was used to compare the statistical difference between groups. The significance of multiple groups was compared using the one-way analysis of variance (ANOVA) followed by the Dunnett’s post hoc test. ANOVA for repeated measurement was performed towards repeated measures data. A value of *P* < 0.05 was considered significant.

## Electronic supplementary material

Supplementary Figure 1

Supplementary Figure 2

Supplementary Figure 3

Supplementary figure legends
