# Supplementary figures and images for: Betulinic acid chemosensitizes breast cancer by triggering ER stress-mediated apoptosis by directly targeting GRP78
Source: Cell Death Dis. 2018 May 25;9(6):636. doi: 10.1038/s41419-018-0669-8 (PMC5970196; doi:10.1038/s41419-018-0669-8)

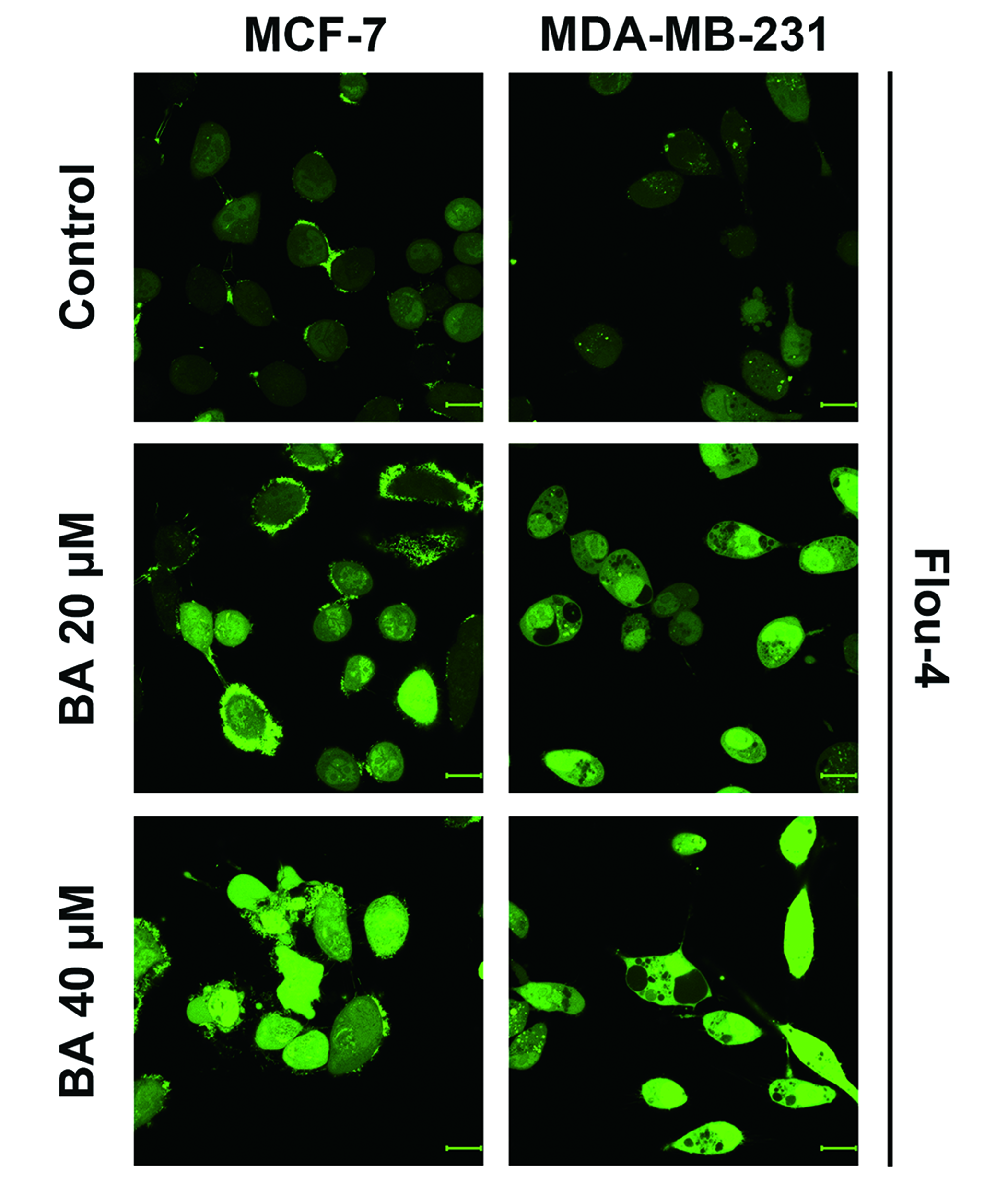

Supplement: Supplementary file 1 — Supplementary Figure 1 [file 41419_2018_669_MOESM1_ESM.tif]

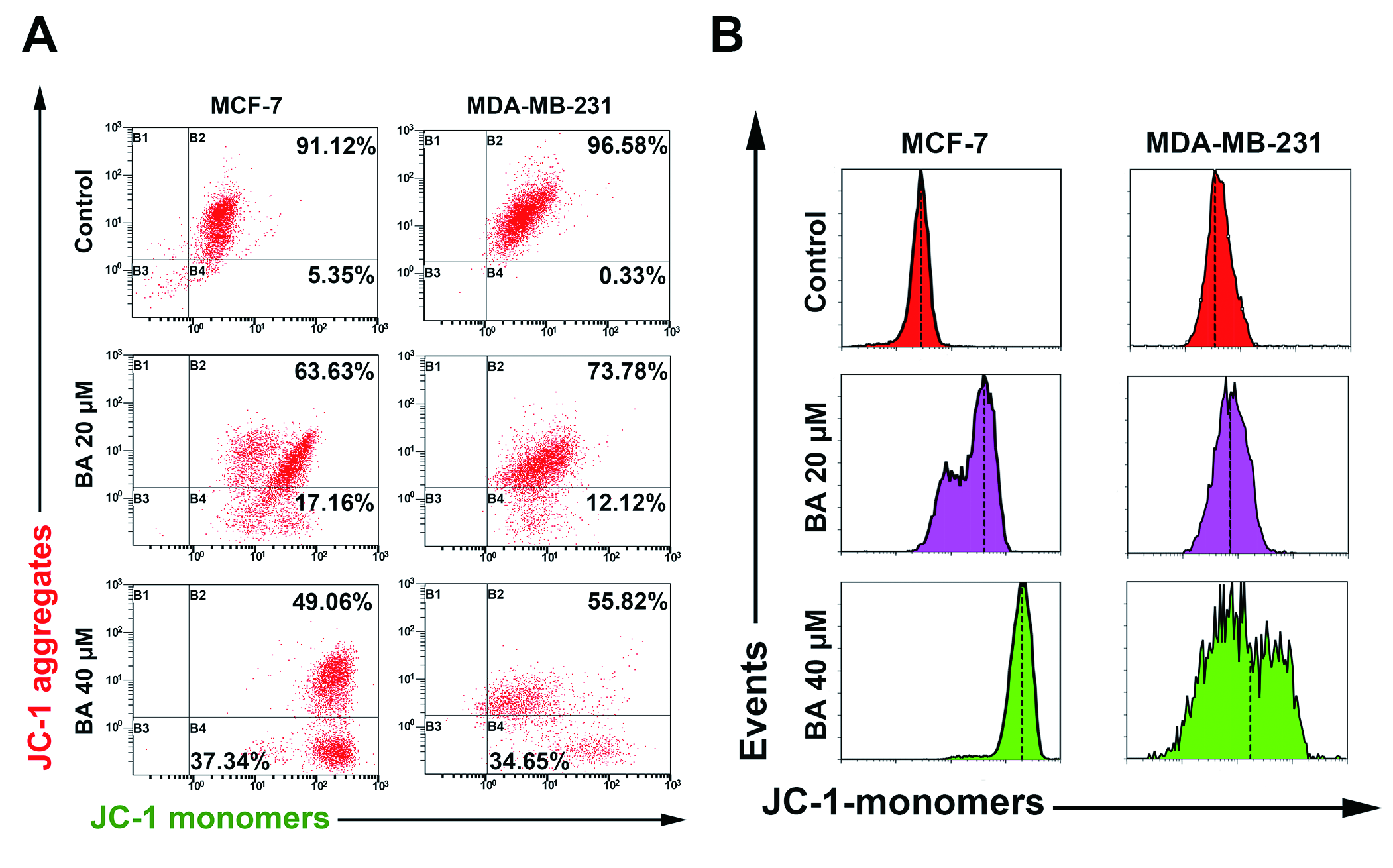

Supplement: Supplementary file 2 — Supplementary Figure 2 [file 41419_2018_669_MOESM2_ESM.tif]

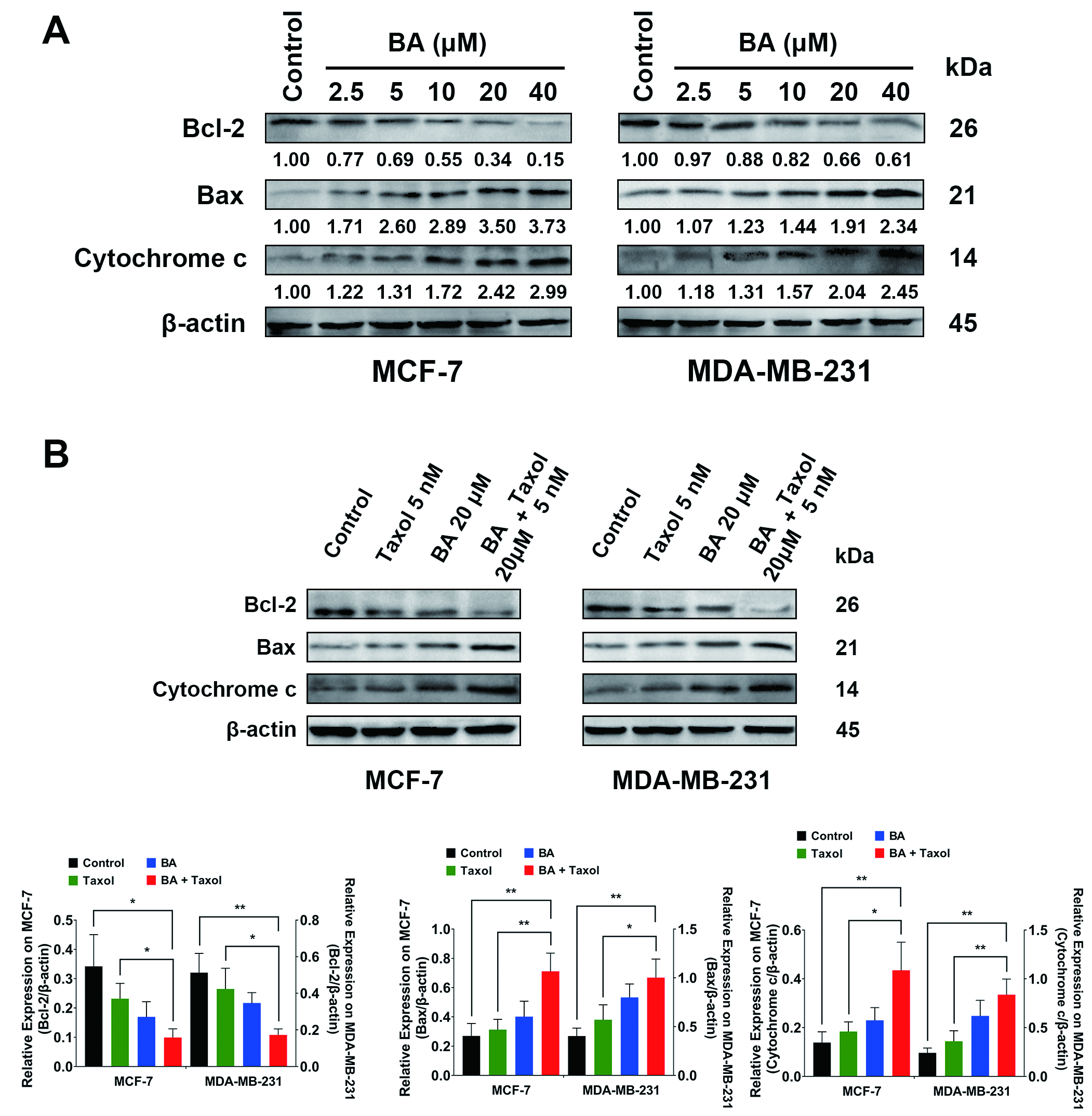

Supplement: Supplementary file 3 — Supplementary Figure 3 [file 41419_2018_669_MOESM3_ESM.tif]
