# Supplementary material for: Betulinic acid chemosensitizes breast cancer by triggering ER stress-mediated apoptosis by directly targeting GRP78
Source: Cell Death Dis. 2018 May 25;9(6):636. doi: 10.1038/s41419-018-0669-8 (PMC5970196; doi:10.1038/s41419-018-0669-8)
Supplement: Supplementary file 4 — Supplementary figure legends [file 41419_2018_669_MOESM4_ESM.docx]

**Supplementary Figure 1. BA elevates intracellular free calcium concentration** **([Ca^2+^]_i_).** The intracellular calcium concentration was determined with Fluo-4/AM staining and detected by laser scanning confocal microscopy. BA elevated the intracellular free calcium concentration in breast cancer cells after BA treatment. Green scale bars indicate 10 μm.

**Supplementary Figure 2. BA induces** **a disruption of mitochondrial transmembrane potential (ΔΨm) in breast cancer cells.** (A) ΔΨm was evaluated by the JC-1 aggregate/JC-1 monomer fluorescence. BA treatment induced the increase of JC-1 monomer accumulation in both breast cancer cells, indicating the mitochondrial membrane potential was shifted from a high ΔΨm to the low ΔΨm. (B) Histogram analysis of green-emitting JC-1 monomer populations in both breast cancer cells following BA treatment.

**Supplementary Figure 3.** **BA triggers mitochondrial pathway apoptosis.** (A) The level of Cytochrome c of breast cancer cells following BA treatment was increased dose-dependently. Accordingly, the expression of Bax was increased, while the anti-apoptotic signal Bcl-2 level was decreased. (B) BA synergistically interacted with Taxol to activate mitochondrial pathway apoptosis, presenting as increased expressions of Cytochrome c and Bax, and the downregulation of Bcl-2 (Values represented as mean ± SD, n=3, **P*< 0.05, ***P*< 0.01).
